# Forest Fragmentation and Selective Logging Have Inconsistent Effects on Multiple Animal-Mediated Ecosystem Processes in a Tropical Forest

**DOI:** 10.1371/journal.pone.0027785

**Published:** 2011-11-16

**Authors:** Matthias Schleuning, Nina Farwig, Marcell K. Peters, Thomas Bergsdorf, Bärbel Bleher, Roland Brandl, Helmut Dalitz, Georg Fischer, Wolfram Freund, Mary W. Gikungu, Melanie Hagen, Francisco Hita Garcia, Godfrey H. Kagezi, Manfred Kaib, Manfred Kraemer, Tobias Lung, Clas M. Naumann, Gertrud Schaab, Mathias Templin, Dana Uster, J. Wolfgang Wägele, Katrin Böhning-Gaese

**Affiliations:** 1 Department of Ecology, Institute of Zoology, University of Mainz, Mainz, Germany; 2 Biodiversity and Climate Research Centre, Frankfurt (Main), Germany; 3 Senckenberg Gesellschaft für Naturforschung, Frankfurt (Main), Germany; 4 Conservation Ecology, Department of Ecology, University of Marburg, Marburg, Germany; 5 Zoological Research Museum A. Koenig, Bonn, Germany; 6 Department of Animal Ecology and Tropical Biology, Biocenter, University of Würzburg, Würzburg, Germany; 7 Animal Ecology, Department of Ecology, University of Marburg, Marburg, Germany; 8 Institute of Botany, University of Hohenheim, Stuttgart, Germany; 9 National Museums of Kenya, Nairobi, Kenya; 10 Biological Collection, University of Bielefeld, Bielefeld, Germany; 11 Department of Bioscience, Aarhus University, Aarhus C, Denmark; 12 Department of Animal Physiology, University of Bayreuth, Bayreuth, Germany; 13 Department of Geomatics, Karlsruhe University of Applied Sciences, Karlsruhe, Germany; 14 Department of Biological Sciences, Goethe University, Frankfurt (Main), Germany; University of Guelph, Canada

## Abstract

Forest fragmentation and selective logging are two main drivers of global environmental change and modify biodiversity and environmental conditions in many tropical forests. The consequences of these changes for the functioning of tropical forest ecosystems have rarely been explored in a comprehensive approach. In a Kenyan rainforest, we studied six animal-mediated ecosystem processes and recorded species richness and community composition of all animal taxa involved in these processes. We used linear models and a formal meta-analysis to test whether forest fragmentation and selective logging affected ecosystem processes and biodiversity and used structural equation models to disentangle direct from biodiversity-related indirect effects of human disturbance on multiple ecosystem processes. Fragmentation increased decomposition and reduced antbird predation, while selective logging consistently increased pollination, seed dispersal and army-ant raiding. Fragmentation modified species richness or community composition of five taxa, whereas selective logging did not affect any component of biodiversity. Changes in the abundance of functionally important species were related to lower predation by antbirds and higher decomposition rates in small forest fragments. The positive effects of selective logging on bee pollination, bird seed dispersal and army-ant raiding were direct, i.e. not related to changes in biodiversity, and were probably due to behavioural changes of these highly mobile animal taxa. We conclude that animal-mediated ecosystem processes respond in distinct ways to different types of human disturbance in Kakamega Forest. Our findings suggest that forest fragmentation affects ecosystem processes indirectly by changes in biodiversity, whereas selective logging influences processes directly by modifying local environmental conditions and resource distributions. The positive to neutral effects of selective logging on ecosystem processes show that the functionality of tropical forests can be maintained in moderately disturbed forest fragments. Conservation concepts for tropical forests should thus include not only remaining pristine forests but also functionally viable forest remnants.

## Introduction

Human disturbance is a major driver of global environmental change [1] and is a threat to biodiversity [2], species interactions and ecosystem processes in tropical forests [3], [4].

Two important drivers of human disturbance are deforestation and forest fragmentation at large spatial scales [4] and selective logging at small spatial scales [5]. At the landscape scale, forest loss and fragmentation severely affect biodiversity [4], [6]. Species richness declines in fragmented forests because remnants may be too small for species to persist or too isolated to be colonised from other remnants [7]. Edge effects and spill-over of species from secondary habitats can also modify the species composition in fragmented forests [8]. Selective logging may either increase or decrease forest biodiversity [5], [9]. At least in moderately logged forests, species communities can be similar to those of undisturbed forests [10], [11], while the impacts of selective logging can strongly change the environmental conditions in tropical forests [12].

Although there is a consensus that human disturbance strongly affects tropical biodiversity [4], many studies have reported that taxa can be affected differently by the same type of human disturbance [13], [14]. Idiosyncratic responses to human disturbance have particularly been found for highly mobile taxa, e.g. for bees and bats [13]. The response to human disturbance varies even among different functional guilds within a taxonomic group [15], [16]. For instance, it is widely documented that insectivorous birds are more susceptible to human disturbance than frugivorous or nectarivorous birds [15], [16]. Owing to such guild- or even species-specific responses, the effects of human disturbance on alpha-diversity (species richness) are usually weaker than on beta-diversity (community turn-over) [13], [14]. Although it is consensus that human disturbance strongly modifies tropical biodiversity, we still know very little about the consequences of changes in species communities for ecosystem processes in tropical forests [4].

A loss of biodiversity can disrupt ecosystem processes [17] but most of our knowledge about the relationship between biodiversity and ecosystem processes originates from small-scale experimental studies [18] that have mostly examined plant-related processes [19]. However, the functioning of real world ecosystems depends on multiple processes [20], and many of these processes involve species interactions across trophic levels [21]. In real world ecosystems, reduced species richness can negatively affect animal-mediated ecosystem processes [22] but the consequences of species loss depend on the functional roles of the species that become extinct [23], [24]. The abundance of functionally important species can therefore strongly influence animal-mediated processes [25]. Thus, not only species richness but also community composition contributes to the relationship between biodiversity and ecosystem processes [22].

Effects of human disturbance on ecosystem processes can also be mediated by changes in resource distributions that strongly influence the spatial distributions of highly mobile organisms such as pollinators [26] and frugivores [27]. Animal movements in response to heterogeneous resource distributions have been shown to strongly affect ecosystem processes [28], [29] and could compensate for species loss in disturbed habitats [30]. To unravel the complex relationships between human disturbance, biodiversity and animal-mediated ecosystem processes, we urgently need studies that simultaneously analyse multiple functional groups and ecosystem processes along human disturbance gradients. Structural equation models provide a valuable tool to study these relationships within a common statistical model and offer the opportunity to disentangle direct environmental from indirect biodiversity-related effects of human disturbance on ecosystem processes [31].

**Figure 1:**
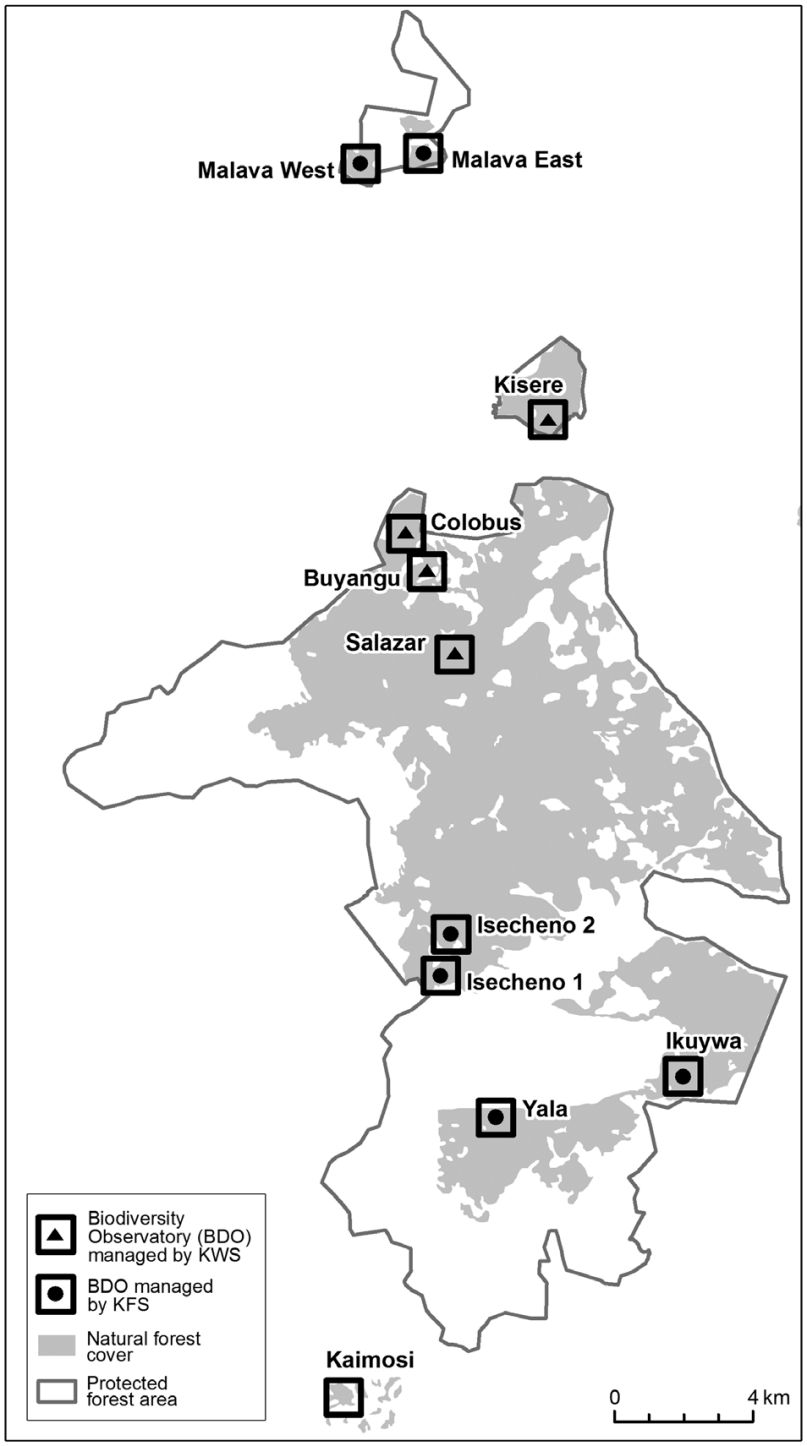
Map of the study area showing the location of the 11 Biodiversity Observatories (BDOs) in Kakamega Forest, Kenya . Note the extension of natural forest cover within the protected forest area (derived from Landsat satellite imagery, 18/05/2003). The squares around each BDO centre are 1 km^2^ in size, but almost all studies were carried out in a 100 m×100 m plot in the centre of each BDO. Management authorities were Kenya Wildlife Service (KWS) or Kenya Forest Service (KFS); Kaimosi is privately owned and similarly managed as KFS sites. Intensities of selective logging in each BDO are provided in Table S1.

Here, we use a comprehensive data set from a Kenyan rainforest in a synthetic analysis of the effects of two drivers of human disturbance, i.e. forest fragmentation and selective logging, on multiple animal-mediated ecosystem processes. In Kakamega Forest, 11 Biodiversity Observatories (BDOs) were established in the main forest block (5 sites) and in each of the adjacent forest fragments (6 sites) (Fig. 1). Forest fragments have been disconnected from the main forest for at least 50 years [32]. During the last 20 years, each BDO has experienced different intensities of moderate selective logging (range: 0–34 trees/ha) owing to different management authorities in different parts of the forest. In this study, we were therefore able to test whether the long-lasting impacts of fragmentation on the forest ecosystem differ from the more recent and localized impacts of selective logging. To do so, we recorded in each BDO the intensities of six ecosystem processes involving plant–animal and animal–animal interactions (pollination, seed dispersal, seed predation, decomposition, army-ant raiding, antbird predation). We stress that we did not quantify ecosystem services, i.e. the goods provided by an ecosystem, but the interactions between different ecosystem components, i.e. ecosystem processes (*sensu* [33]). Interactions between ecosystem components are, for instance, those between plants and their pollinators (mutualistic) or between prey species and their predators (antagonistic) [1], all of which contribute to the functionality of an ecosystem [17]. To relate ecosystem processes to biodiversity, we also collected data on species richness and community composition for the six animal taxa involved in these processes. Thus, we were able to investigate the effects of the two different drivers of human disturbance on multiple ecosystem processes and on two components of biodiversity (i.e. species richness and community composition). We address two main hypotheses: (1) Forest fragmentation and selective logging modify ecosystem processes and alter species richness and community composition in Kakamega Forest. (2) Indirect effects of human disturbance mediated by changes in biodiversity strongly affect ecosystem processes. To test the two hypotheses, we quantified the effects of forest fragmentation and selective logging on ecosystem processes and biodiversity of related functional groups, and constructed structural equation models to disentangle biodiversity-related indirect effects from direct effects of human disturbance.

## Materials and Methods

### Ethics Statement

Kenya Wildlife Service (KWS), Kenya Forest Service (KFS) and private land owners issued all necessary permits for the work conducted in Kakamega Forest.

### Study area

This study was carried out in Kakamega Forest in western Kenya (0°07′–0°27′ N, 34°46′–34°57′ E). Kakamega Forest is a montane tropical rainforest at 1,500–1,700 m asl and is highly fragmented and locally disturbed [32]. The forest consists of a main block covering approximately 9,500 ha and six forest fragments of various sizes (40–1,348 ha; Table S1, Fig. 1); the forest is managed by KWS or KFS [32]. A densely populated agricultural area (643 people per km^2^ in 1999) surrounds the forest, resulting in high demands of the human population on forest resources (e.g. on firewood and timber trees). Owing to protective measures since the early 1980s, disturbance intensities have been low in the highly protected areas (managed by KWS) and moderate in the less protected areas (managed by KFS) during the last decades [32], [34] (Table S1). In 2001, 11 study sites (Biodiversity Observatories, BDOs) were established in Kakamega Forest. Three are located in the northern part and two in the southern part of the main forest, and one in each of the six fragments (Fig. 1). At each site, field studies were conducted in a core area of 100 m×100 m around the BDO centre except for the inventories of selective logging and of army ants where the study transects extended beyond this core area. All data presented here were collected in at least 9 of the 11 BDOs between 2002 and 2009.

### Human disturbance

To quantify human disturbance in the BDOs, we assessed the size of each forest fragment and the intensity of selective logging. Forest size was derived from visual interpretation of 2003 Landsat imagery. Forest size was the same for the five BDOs situated in the main forest (Table S1). We could not replicate our studies in different main forest blocks because only a single large forest block has remained in the study area. Still, different BDOs in the main forest block are reasonable replicates because the main forest is not structurally homogeneous and different authorities manage the northern and the southern part of the forest (Fig. 1, Table S1). We did not incorporate the distances between fragments and the main forest into our analyses but tested the effect of forest fragmentation only in terms of forest size. We additionally determined the distance of each BDO centre to the nearest forest edge from visual interpretation of 2003 Landsat imagery. The distance to the forest edge did not affect any of the measured ecosystem processes and components of biodiversity and was therefore not considered in further analyses. To quantify the degree of local disturbance, we assessed the intensity of selective logging as the number of trees logged per ha. In a previous study, this indicator was the best to quantify differences in local-disturbance intensities in Kakamega Forest [34]. Nevertheless, other human impacts that are more difficult to assess, such as hunting, may also differ between differently disturbed areas of Kakamega Forest [34]. Inventories of selective logging were conducted in 2002 and 2003 in each BDO by walking several transects through the forest that summed to a length of at least 1,000 m (except for Kaimosi: 280 m [34]). To differentiate between recent and past selective logging, the approximate time since cutting was estimated by examining the degree of decomposition of the remaining stump [34]. In our analyses, we used the estimated number of logged trees with a DBH >10 cm during the last 20 years. Selective logging ranged from very low (0 trees/ha) to moderate intensities (34 trees/ha, Table S1); selective logging also varied between the BDOs in the main forest block (0–13 trees/ha), due to different management authorities in different parts of the main forest (Table S1).

### Ecosystem processes

In at least nine BDOs, we assessed six animal-mediated ecosystem processes, i.e. (i) pollination, (ii) seed dispersal, (iii) seed predation, (iv) decomposition, (v) army-ant raiding and (vi) antbird predation (see Table S1 for a list of processes studied in each BDO).

(i) We assessed pollination of flowering plants of *Justicia flava* (Acanthaceae) in 10 BDOs (excluding Malava West) from November 2008 to January 2009. We deliberately chose this plant species as representative model species, because it is widespread in the understory of different forest habitats [35]. Many different bee species have been recorded to forage on the yellowish nectar-rich flowers of *J. flava,* which is not self-pollinating and produces short-lived two-day flowers [35]. In the centre of each BDO, we established three artificial patches of 1 m^2^ with flowering *J. flava* plants. At least one week prior to the experiment, we collected flowering plants outside the BDOs and transferred 50 plants per patch into five water-filled plastic tubes that were regularly spaced at the four corners and in the centre of each patch. After five days, we randomly collected 10 open flowers in each patch (i.e. 30 flowers per BDO), cut off the stigmas and placed them into ethanol-filled plastic tubes. Later, pollen grains deposited on the stigma were counted at 60-times magnification under a stereomicroscope (M3C, Wild, Heerbrugg, Switzerland), and the number of conspecific pollen grains on the inner sides of the stigmatic lobes was used as a measure of pollen deposition by insects.

(ii) Seed dispersal by frugivorous birds was estimated from a case study with *Prunus africana* (Rosaceae) [36], a widespread tree in tropical Africa and in Kakamega Forest. In March 2002, October 2002, March 2003, and December 2003, we observed all fruit-eating birds on a total of 28 trees (1–5 trees per BDO) in the centre of each BDO (excluding Salazar and Malava West). Each tree was observed once between 07:00 and 19:00 hours. Every 30 min (07:00, 07:30, 08:00 hours, etc.), all fruit-eating birds in the tree were recorded for a period of 1 min; seed-removal rates of frugivorous birds were recorded in between the 30-min intervals. For each tree and bird species, we summed the number of individuals over all 25 1-min observations and multiplied this number with the mean seed-removal rate per bird species [36]. We summed for each tree the number of seed-removal events for the 24 bird species that were observed to swallow seeds and thus possibly contributed to seed dispersal. We used the mean number of seed-removal events per tree as a measure for seed dispersal in each BDO. We were not able to assess the actual seed-dispersal distances provided by the frugivorous birds.

(iii) We experimentally assessed predation rates of seeds of *P. africana* in nine BDOs (excluding Malava West and Salazar) in the dry and the wet season of 2003 and 2006 [37]. In the centre of each BDO, three transects were established. During each survey, three plastic dishes (5 mm depth, 120 mm diameter), each with one seed of *P. africana*, were placed along each transect. Seed fate was monitored for two consecutive nights. We replaced seeds that had disappeared or had been eaten during the first night. Seeds were classified as having been eaten when seed remnants were found on or close to the dishes. Seed predation was calculated as proportion of seeds depredated per night and BDO [37]. For each BDO, we calculated the mean predation rate over seasons and years.

(iv) We investigated decomposition rates of organic leaf matter by the leaf litter fauna in all BDOs from December 2008 to February 2009. For decomposition experiments, we chose *Croton* spp. that are widespread and abundant tree species in Kakamega Forest. In the centre of each BDO, we exposed 20 *Croton* leaf samples equally distributed in mesh bags of small (20×20 µm) and large (5×5 mm) mesh width to differentiate between decomposition by the microfauna and by the leaf litter macrofauna. The original mass of each *Croton* leaf was weighed, and mesh bags were then exposed on the forest floor in the centre of each BDO. After two months, bags were collected, and leaves were washed, dried, and weighed again. The decomposition rate was calculated as the proportional weight loss of leaf material over time. We subtracted the decomposition rates in small-meshed bags (excluding macrofauna) from those in large-meshed bags (including macrofauna) to determine the decomposition by the macrofauna because we aimed at analysing the relationship between the community composition of the leaf litter macrofauna and their contribution to decomposition. Contributions to decomposition by the leaf litter macrofauna can be positive or negative because the macrofauna influences decomposition rates directly by decomposing leaf matter and indirectly by grazing on the decomposing microfauna [38].

(v) Since army-ant raiding profoundly affects invertebrate prey populations in tropical forests [39], we measured raiding rates of the two co-occurring army ant species *Dorylus wilverthi* and *Dorylus molestus* from April 2004 to August 2005. In each BDO, we established three 500-m transects and placed six pitfall traps (diameter: 75 mm) along each transect [40]. Pitfall traps were checked weekly for the presence of the swarm-raiding army ants *D. wilverthi* and *D. molestus* for a total of 19 weeks. The raiding rate was calculated as the number of pitfall trap checks in which army ant individuals of the respective species were found divided by the total number of checks of pitfall traps on each transect. In cases, where workers of both species were found within one trap, two raiding events were counted. This measure estimates the frequency with which a random point in the forest has been raided by army ants and is a good estimate of predation activity and intensity of army-ants in a BDO because army ants prey upon large amounts of animal prey along their raiding paths [39].

(vi) To assess the intensity of predation by ant-following birds, we repeatedly searched for army-ant swarm raids in all BDOs from April 2004 to August 2005 [41]. We found no raids in Buyangu and encountered 1–17 raids in the other BDOs. When a swarm raid was located, all bird activity was observed in its surroundings for 60 min. Ant-following bird species were defined as those bird species taking up prey directly at swarm raids. Species were identified by both sight and sound, and the maximum number of simultaneously observed individuals of each bird species was recorded as an estimate of species abundance. To estimate predation activity of ant-following birds at the swarm raids, we determined for each bird species its body mass [42] and calculated the fresh matter intake per hour for each bird individual using an allometric relationship for insectivorous birds [43]. For each BDO, we averaged the estimates of fresh matter intake per bird flock over repeated observations on different raids. Note that this estimate of antbird predation is strongly determined by the size-distribution of birds in the antbird flocks and is not related to the overall antbird abundance in a BDO (*n* = 10, *r* = 0.03, *P* = 0.927).

### Biodiversity

According to its definition (e.g. [17]), biodiversity encompasses not only the number of species (i.e. species richness) but also the relative abundance and species composition of a community (i.e. community composition). We therefore recorded both species richness and community composition of the six animal taxa or functional guilds that were involved in the respective processes, i.e. (a) bees, (b) frugivorous and ant-following birds, (c) rodents, (d) decomposing leaf litter macrofauna, and (e) army ants. (a) In ten BDOs (excluding Malava West), we caught bees with pan traps (plastic dishes of 750 mL) which had been sprayed with white UV-colour. From November 2008 to January 2009, three pan traps per BDO were exposed on the forest floor for five days. Two trapping sessions were carried out in each BDO. Caught bees were stored in ethanol-filled plastic tubes. Bees were determined to morphospecies according to identification keys [44], [45] and reference collections at the National Museums of Kenya. The number of species per BDO was determined, and cumulative numbers of individuals per species were used for ordination analysis.

(b) In each of the 11 BDOs, a single observer conducted 10-min bird point counts at 18 point count stations between 07:00 and 11:00 hours [41]. In 2004 and 2005, each point count station was visited six times, and a total of 1,176 point counts were carried out. At each point count station, all bird species seen or heard within a radius of 25 m were recorded. For each BDO, we determined the number of species and the cumulative number of individuals per species. The bird community was partitioned into ant-following and frugivorous birds. Bird species were classified as ant-followers according to observations at army-ant raids [41]; bird species were classified as frugivores according to [46].

(c) We trapped small mammals on the forest floor in the dry and rainy season in 2003 and 2006 in nine BDOs (excluding Malava West and Salazar). We set up 99 Sherman live-traps (98×115×295 mm, baited with peanut butter) along nine transects of 100-m length in each BDO [37]. Neighbouring transects were separated from each other by 10 m. For both seasons, trapping was conducted for three consecutive nights in each plot. Trapped rodents were identified to species level (according to [47]). For each BDO, we determined the species number and the cumulative abundance of each species caught over the two study years and seasons.

(d) From August to November 2005, 12–18 samples of leaf litter were collected at 4–6 sites in each of the 11 BDOs. To standardize sampling, an acrylic frame (height 25 cm, area 0.25 m^2^) was laid on the forest floor, and all leaf litter within the frame was transferred to a sifter (mesh size: 10×10 mm); large insects were added by hand. Invertebrates were extracted from the sifted leaf litter using the Winkler extraction method. Two to three mesh bags with a mesh size of 4 mm were filled with leaf litter and were kept for seven to nine days in a closed Winkler bag. During this time, the leaf litter dried, causing invertebrates to leave the mesh bags and to fall into an ethanol-filled plastic cup at the base of the bag. Winkler extraction was followed by hand-collecting remaining individuals from the leaf litter. For each BDO, we determined the cumulative number of individuals for the following taxa that are typical decomposers [38], [48]: Acari, Annelida, Apterygota, Coleoptera (without Staphylinidae), Gastropoda, Isopoda, Isoptera, and Myriapoda. Identification to species level was not feasible.

(e) Abundance of the two swarm-raiding army ant species was measured from April 2004 to December 2005 in all 11 BDOs. In each BDO, three 500-m transects were established. Each transect was monitored approximately 40 times in the mornings by searching for foraging trails, emigration trails, and raids of army ants [40]. Observations of trails and raids within 100-m distance were defined as belonging to the same colony. We estimated the abundance of the two species as the total number of colonies per transect kilometre.

### Data analysis

In all analyses, we multiplied forest size by −1 so that the effects of human disturbance (forest fragmentation, selective logging) increase from low to high values. Forest size and the intensity of selective logging were log-transformed and were not significantly correlated (*n* = 11, *r* = 0.38, *P* = 0.245). Estimates of ecosystem processes were analysed on a logarithmic (pollination, seed dispersal, antbird predation) or angular scale (seed predation, army-ant raiding); estimates of decomposition were not transformed. To investigate the effects of forest size and of selective logging on ecosystem processes, we fitted five linear models, i.e. (i) both main effects and interaction term, (ii) both main effects, (iii) forest size, (iv), selective logging, (v) only intercept. For each process, we selected a single minimal adequate model according to the corrected Akaike information criterion (AICc). To test for potential effects of spatial autocorrelation, we calculated Moran's *I* values from the residuals of all minimal adequate models based on a spatial weights matrix derived from the neighbourhoods of each BDO [49]. The neighbourhood of each BDO was defined by the four nearest BDOs. Significance of Moran's *I* values was tested with a permutation test (1,000 permutations). Tests of spatial autocorrelation resulted in qualitatively equal results when only the two nearest BDOs were considered.

Furthermore, we determined the overall effect sizes across all ecosystem processes for each disturbance variable and tested the heterogeneity of the responses to human disturbance by carrying out a random-effects meta-analysis using the DerSimonian-Laird (DSL) approach [50]. Random-effects models allow more general inferences and are better suited for ecological data affected by both sampling error and random variation [51]. As suggested for meta-analysis [51], correlation coefficients between the respective ecosystem process and forest size and selective logging were *z*-transformed and weighted by their sample size (the number of BDOs). Differences in the effect sizes between forest size and selective logging as well as the residual heterogeneity in a model were tested with Cochran’s *Q* test [52].

We tested effects of forest size and selective logging on observed species richness (square-root-transformed) and community composition by identifying minimal adequate linear models as described above; we tested for spatial autocorrelation in the residuals of the minimal adequate models. Effects on species richness of army ants (only two species) and leaf litter fauna (no species data available) were not tested. To investigate gradients in community composition, we carried out non-metric multi-dimensional scaling (NMDS) on Bray-Curtis distances of the species abundances in each BDO and a Principal Component Analysis (PCA) based on the correlation matrices of the respective community data [53]. Prior to analyses, measures of species abundance were square-root-transformed. Abundances of leaf litter fauna were analysed at the order level and were Wisconsin-standardised because of a high variation in abundances among orders. NMDS analyses were carried out with two axes (stress <10 in all cases). We tested whether site configurations in NMDS plots were related to forest size and selective logging by projecting environmental vectors onto the ordination plot and by testing their significance with a permutation test (1,000 iterations). We additionally extracted site (BDO) scores of both axes and tested whether forest size and selective logging were related to community turn-over along the first and second axis, respectively. In all taxa, the scores of the second axis were not related to forest size or selective logging. The scores from the first NMDS axis were very closely correlated with the first principal component of the PCA (*r*>0.9); the only exception was the bee community, where both NMDS scores and principal components were not related to forest size or selective logging.

Finally, we used structural equation models (SEMs) to disentangle the direct effects of human disturbance from the indirect biodiversity-related effects on ecosystem processes.

SEMs are a statistical tool to identify potential causal relationships between a set of variables [31]. Model fitting is based on a path diagram that predefines potential causal links within the set of variables. By partitioning the correlation between the variables, effects can be disentangled into direct effects (those between consecutive variables in the diagram) and indirect effects (those mediated by the intermediate variables). The strength of the effects is measured as regression coefficients, here called path coefficients. In this study, we included only those paths into the path diagram that had been identified to be important in prior linear- model analyses; this procedure avoided overfitting of the models given the limited number of study sites [54]. In the models, forest size and selective logging were assigned as uncorrelated. Path models were fitted in AMOS 17.0 (SPSS Inc., Chicago, IL, USA) using maximum-likelihood estimates of path coefficients and their significance. Significance of path coefficients was also tested with a parametric bootstrap technique (1,000 iterations) that provides more conservative estimates of significance. These estimates confirmed the significance of coefficients based on maximum-likelihood estimates (Table S2).

## Results

### Effects of human disturbance on ecosystem processes

**Figure 2:**
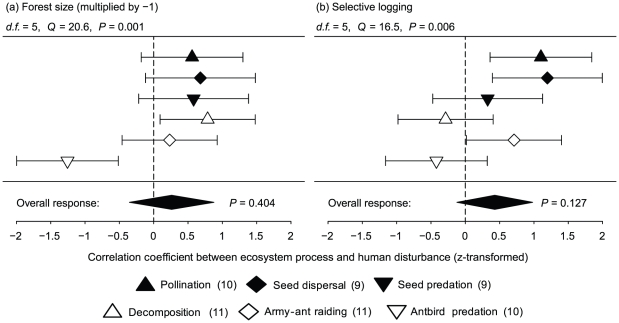
Effect sizes of six ecosystem processes in response to (a) forest size and (b) selective logging in Kakamega Forest, Kenya. Human disturbance increases from low to high values (forest size was multiplied by −1). Given are *z*-transformed correlation coefficients and their 95% confidence intervals for all pair-wise correlations between the respective ecosystem process and forest size and selective logging, respectively. The number of study sites for each process is indicated in parentheses. Overall effect sizes from a random-effects models (DSL approach) are displayed as diamonds. Residual heterogeneity was tested with Cochran's *Q*-test.

All ecosystem processes except seed predation by rodents were affected by forest size or selective logging (Table 1). Decomposition by the leaf litter fauna was higher in small forests, while antbird predation decreased in small forests (Table 1); the other processes were not influenced by forest size. Pollination, seed dispersal and army-ant raiding increased with the intensity of selective logging (Table 1), while the other processes were unaffected. Spatial autocorrelation was not detected in any minimal adequate model (Table 1). The meta-analysis formally confirmed that different ecosystem processes responded differently to the effects of forest size (*df* = 5, *Q* = 20.6, *P* = 0.001) and selective logging (*df* = 5, *Q* = 16.5, *P* = 0.006). Overall effect sizes across all ecosystem processes were slightly positive for forest size and selective logging, but did not differ significantly from zero (Fig. 2). In a combined model of both disturbance types, effect sizes did not differ between forest size and selective logging (*df* = 1, *Q* = 0.16, *P* = 0.689).

**Table 1:** Effects of forest size and selective logging on six ecosystem processes in Kakamega Forest, Kenya.

Ecosystem process	Sites	Disturbance	*r^2^*	β	*t*	*P*	Moran's *I*	*P*
Pollination	10	Logging	0.642	0.801	3.784	0.005	−0.216	0.728
Seed dispersal	9	Logging	0.693	0.832	3.972	0.005	−0.046	0.247
Seed predation	9	No effects	-	-	-	-	−0.130	0.443
Decomposition	11	Forest size	0.761	0.895	4.784	0.001	−0.243	0.860
		Logging		−0.623	−3.329	0.010		
Army-ant raiding	11	Logging	0.373	0.611	2.314	0.046	−0.315	0.985
Antbird predation	11	Forest size	0.722	−0.850	−4.561	0.002	−0.112	0.389

Given are regression parameters from minimal adequate linear models. Human disturbance increases from low to high values (forest size was multiplied by −1). Seed predation was not affected by any disturbance variable. Spatial autocorrelation was assessed by Moran's *I* values, derived from the model residuals and a spatial weights matrix from the four nearest neighbours of each BDO.

### Effects of human disturbance on biodiversity

Human disturbance hardly affected species richness. The only significant effect was an increase in bee species richness in small forests (Table 2). However, community composition of four of six taxa (leaf litter fauna, army ants, antbirds, frugivorous birds) changed substantially in response to forest size (Table 2, Fig. S1); only bee and rodent communities were not affected. Effects of forest size on community composition were mostly driven by differences in community composition between small forest fragments on the one hand and large fragments and main forest on the other hand (Fig. S1). In contrast to the strong effects of forest size, selective logging did not affect species richness or community composition of any taxonomic group. Spatial autocorrelation was absent from all minimal adequate models (Table 2).

**Table 2:** Effects of forest size and selective logging on species richness and community composition of six animal taxa in Kakamega Forest.

Taxon	Sites		Disturbance	*r^2^*	β	*t*	*P*	Moran's *I*	*P*
Leaf litter fauna	11	Species richness:	*Not tested*						
		Community composition:	Forest size	0.462	0.680	2.779	0.021	−0.126	0.513
Army ants	11	Species richness:	*Not tested*						
		Community composition:	Forest size	0.589	0.767	3.590	0.006	0.076	0.115
Understory bees	10	Species richness:	Forest size	0.452	0.672	2.569	0.033	−0.257	0.853
		Community composition:	No effects	-	-	-	-		
Rodents	9	Species richness:	No effects	-	-	-	-		
		Community composition:	No effects	-	-	-	-		
Ant-following birds	11	Species richness:	No effects	-	-	-	-		
		Community composition:	Forest size	0.740	0.860	5.054	<0.001	−0.208	0.735
Frugivorous birds	11	Species richness:	No effects	-	-	-	-		
		Community composition:	Forest size	0.613	0.783	3.774	0.004	−0.229	0.832

Community composition was quantified from non-metric multi-dimensional scaling (NMDS) (site scores on first axis). Given are regression parameters of minimal adequate models. Human disturbance increases from low to high values (forest size was multiplied by −1). As effect directions in NMDS are arbitrary, effects on community composition are given as positive values. Species richness of leaf litter fauna (no species data) and army ants (only two species) were not tested. Spatial autocorrelation was assessed by Moran's *I* values, derived from the model residuals and a spatial weights matrix from the four nearest neighbours of each BDO.

### Relationships between human disturbance, biodiversity and ecosystem processes

**Figure 3:**
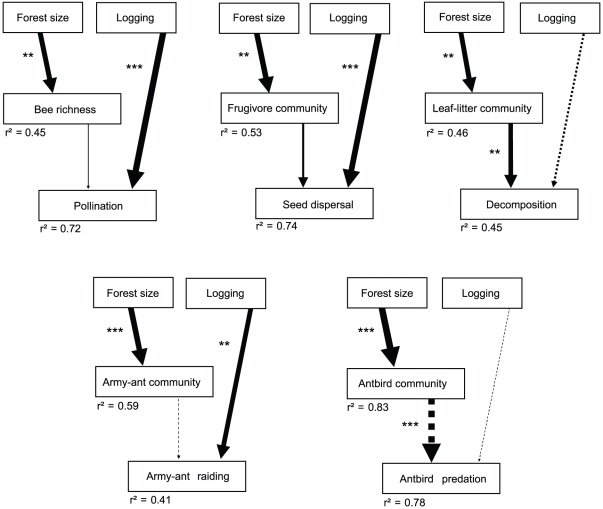
Path models of the indirect effects of forest size and the direct effects of selective logging on five ecosystem processes. Human disturbance increases from low to high values (forest size was multiplied by −1). Biodiversity effects were tested as follows: observed species richness (bees) and site scores of the first NMDS axis (frugivores, leaf litter fauna, army ants, antbirds); the relationships between human disturbance and community composition are by default positive. Arrow width is proportional to path coefficients; continuous arrows show positive, and dotted arrows negative effects. Asterisks indicate significance of path coefficients from maximum-likelihood (ML) estimates. The proportion of explained variance *r^2^* is given for each response variable. Table S2 provides the number of study sites, exact path coefficients and *P*-values from ML and bootstrap estimates. *, *P*<0.05; **, *P*<0.01; ***, *P*<0.001.

We applied structural equation modelling to disentangle direct from biodiversity-related indirect effects of human disturbance on ecosystem processes. Because selective logging did not influence any measured components of biodiversity, effects of selective logging were assigned to be direct and not related to changes in biodiversity. Forest size affected species richness (bees) or community composition (frugivores, leaf litter fauna, army ants, antbirds) and therefore we tested whether forest size influenced ecosystem processes indirectly through changes in species richness or in community composition; note that community composition cannot increase or decrease but can only differ more or less strongly between BDOs. We found that decomposition and antbird predation were influenced by changes in the respective animal communities (leaf litter fauna, antbirds) in differently sized forests (Fig. 3, Table S2). Decomposition was higher in small forests, while predation by antbirds was lower in small forests. In contrast, pollination, seed dispersal and army-ant raiding were not influenced by changes in species richness or community composition, but increased directly with the intensity of selective logging (Fig. 3).

## Discussion

### Effects of human disturbance on ecosystem processes

In Kakamega Forest, all processes except antbird predation increased either in response to forest fragmentation or to selective logging. Hence, the overall effect sizes of forest fragmentation and of selective logging on ecosystem processes were positive, albeit not significantly. In Kakamega Forest, intensities of human disturbance range from low to moderate levels; the smallest forest fragments are still rather large (>40 ha) and the most disturbed sites have only been moderately logged during the last 20 years (<35 trees/ha). This shows that moderate levels of human disturbance do not disrupt the majority of animal-mediated ecosystem processes, at least in Kakamega Forest. A high conservation value of disturbed forests has also been found in other tropical regions [10], [55], given that intensities of human disturbance are moderate and that undisturbed sites occur in the vicinity [55].

Forest fragmentation resulted in an increase in decomposition and in a decrease in antbird predation, whereas selective logging increased pollination, seed dispersal and army-ant raiding in Kakamega Forest. The formal meta-analysis confirmed that the consequences of forest fragmentation and selective logging strongly differed for the particular ecosystem processes. Because all processes were studied along the same disturbance gradient, the differential responses were not caused by differences in environmental context [29], but by the idiosyncratic response of ecosystem processes to human disturbance. This is an important finding for ecosystem research because only few studies analysed multiple ecosystem processes in a single real world ecosystem [56]–[58]. The idiosyncratic response of ecosystem processes to human disturbance suggests that community-level responses to human disturbance are very difficult to predict and will be further complicated by potential feedbacks across trophic levels [1].

### Effects of human disturbance on biodiversity

Forest fragmentation strongly affected community composition in Kakamega Forest, whereas the intensity of selective logging did not influence any taxonomic group. The strong differences in community composition between large and small forest fragments are congruent with the patterns found in Amazonia, where leaf litter fauna, army ants, and understory birds strongly respond to fragment size [59]. In contrast to the Amazonian study [6], fragmentation of Kakamega Forest did not reduce species richness. The relatively large size of the forest fragments (>40 ha) may mitigate the effects of forest fragmentation on species richness. In comparison, the smallest fragments established in the Amazonian fragmentation experiment are only 1 ha in size [6]. Another factor alleviating the effects of forest fragmentation on species richness could be the structurally rich farmland surrounding Kakamega Forest [35], [60]. Spill-over of species from the species-rich farmland into forest fragments may explain the increase in bee species richness in small forest fragments [35] and the high species turn-over of frugivorous birds [60]. In contrast to the strong effects of fragmentation, the moderate intensities of selective logging in Kakamega Forest did not affect species richness or community composition. This is consistent with review studies from tropical forests [5], [9] and recent findings from Bornean rainforests [10], [11].

### Relationships between human disturbance, biodiversity and ecosystem processes

Changes in biodiversity can alter ecosystem processes in real world ecosystems [22], but depending on the environmental context [29] changes in animal behaviour can be more important for animal-mediated processes than changes in biodiversity [28]. In Kakamega Forest, we found that the effects of human disturbance on ecosystem processes were highly idiosyncratic because of different mechanisms involved in the responses of the respective processes.

Two of five processes, decomposition and antbird predation, were presumably affected by changes in community composition related to forest fragmentation. In Kakamega Forest, the leaf litter fauna differed between differently sized forests which was correlated with higher decomposition rates in small forests. This could be due to a higher abundance of isopods in small forests (mean number of isopods sampled per BDO: 17 individuals in the main forest vs. 41 individuals in the small forest fragments) because isopods can increase decomposition rates by litter fragmentation and stimulation of microbial activity [38]. In contrast to decomposition, predation by antbirds strongly decreased in small forests. We explain the decline in predation rates with the absence (*Neocossyphus poensis* [White-tailed ant-thrush], 52 g body mass) or strongly reduced abundance (*Bleda syndactyla* [Red-tailed bristlebill], 46 g body mass) of two large-bodied and highly specialised ant-following species from small forest fragments [41], [61]. A much smaller and less-specialised species (*Sheppardia polioptera* [Grey-winged robin], 17 g body mass) that increased in abundance in the small forest fragments was not able to compensate the loss of these functionally important species [41], [61]. This supports the idea that a decline of species contributing unique functional traits to a community strongly reduces ecosystem processes [22], [62].

In Kakamega Forest, selective logging directly affected three ecosystem processes, all of them positively. According to previous studies, selective logging strongly affects forest structure [63] and microclimate [12], and can modify resource availabilities [64]. In our system, selective logging indeed increased canopy openness (data from hemispherical photographs: *n* = 10, *r* = 0.64, *P* = 0.048) and strongly reduced vertical foliage height diversity (*n* = 10, *r* = −0.79, *P* = 0.006; C. Mammides, unpublished data). It is very likely that these changes in forest structure also affected the availability as well as the spatial distribution of resources (e.g. of flowers, fruits and invertebrates) at sites with different intensities of selective logging. It has been proposed that a lower availability or clumped distribution of fruits at disturbed sites increases the attractiveness of fruiting trees at selectively logged sites in Kakamega Forest [36]. In disturbed habitats, highly aggregated resources such as remnant fruiting trees generally attract frugivorous birds [65] and thus are important seed-dispersal foci that help to maintain ecological processes in degraded habitats [27]. A very similar effect of resource heterogeneity could explain the increase in pollen deposition on experimental flower arrays at selectively logged sites because a temporary aggregation of bee pollinators on flower patches occurs if other floral resources are rare or not homogeneously distributed in the surroundings [28]. Differences in army-ant raiding between sites of different intensities of selective logging could similarly be caused by different resource availabilities on the forest floor. At logged sites, a higher solar radiation on the forest floor may increase the structural complexity of the understorey vegetation [63] and thus invertebrate prey and predator densities [66]. The overall density of army-ant colonies, however, only slightly increased with the intensity of selective logging in Kakamega Forest (*n* = 11, *r* = 0.45, *P* = 0.165). This suggests that the positive effect of selective logging on army-ant raiding was not merely an effect of increased army-ant abundances but might also be due to behavioural changes of army ants, e.g. changes in foraging ranges, in response to altered resource availabilities. Distinct microclimates at logged sites could also affect the raiding behaviour of army ants because one of the two army ant species occurring in Kakamega Forest (*Dorylus molestus*) is influenced by the microclimatic conditions in the forest understorey [61].

We are aware that the correlative approach of our analyses and the lack of resource data for the BDOs make it impossible to identify the ultimate mechanisms driving the direct effects of selective logging on ecosystem processes. Potentially, these effects could also be the result of undetected changes in biodiversity at selectively logged sites. However, this appears to be unlikely because the strong changes in biodiversity for bees, birds and army ants in forest fragments were not related to changes in the respective ecosystem processes. The high mobility of the involved taxonomic groups, i.e. of pollinating bees [67], frugivorous birds [30] and army ants [68], rather indicates that the positive effects of selective logging on ecosystem processes were not caused by changes in biodiversity but by changes in animal behaviour in response to altered environmental conditions at selectively logged sites. In comparison to the high mobility of these taxonomic groups, insectivorous birds [15] and leaf litter fauna forage at small spatial scales and are unlikely to cross habitat borders. Therefore, changes in their biodiversity are more likely to be translated into changes in the respective ecosystem process.

### Caveats

In this study, we synthesized data across various case studies that investigated different ecosystem processes and functional groups. Such studies are very important for a more general understanding of the effects of human disturbance on species interactions and ecosystem processes [1]. However, we are aware that our study still has its limitations in terms of generality. We were only able to use model species (and not the entire species community) to assess four of six ecosystem processes (pollination, seed dispersal, seed predation, decomposition). To minimize this limitation, we chose model species that are widespread and abundant in the forest and thus might be representative for the entire species community. Nevertheless, future studies assessing ecosystem processes by considering more species or entire communities are desirable and would strengthen the inferences made from our study. Because we were primarily interested in the spatial variation in ecosystem processes and biodiversity in Kakamega Forest, we maximized our sampling efforts to cover as many BDOs as possible, thereby constraining sampling intensities within each BDO, for instance for assessing temporal fluctuation. However, sampling intensities per BDO were sufficiently high to reasonably quantify the differences in ecosystem processes and biodiversity among BDOs. Still, our sampling design only included 11 sites because the number of forest remnants and their accessibility made it impossible to include more sites. One consequence of the small number of replicate sites was that we were unable to fit more sophisticated path models to our data set. Fortunately, the effects of forest fragmentation and selective logging on both ecosystem processes and community composition in Kakamega Forest were so strong that we were able to reveal strong impacts of human disturbance on ecosystem functioning, even though our sample size was small.

### Conclusions

We found that ecosystem processes respond in distinct ways to forest fragmentation and selective logging in Kakamega Forest. The impact of human disturbance on ecosystem processes ultimately depended on both the response of a process to changes in biodiversity and to changes in environmental conditions. Our findings strongly suggest that forest fragmentation primarily affects ecosystem processes that are sensitive to changes in the community composition of the involved organisms. In contrast, ecosystem processes mediated by animal communities with highly mobile species may respond primarily to changes in local forest structure and resource distributions, for instance caused by selective logging. These intricate findings call for more studies in real world ecosystems that simultaneously analyse the responses of multiple functional groups and ecosystem processes to different drivers of global environmental change. From a conservation perspective, the positive to neutral effects of selective logging on ecosystem processes, even in forest fragments, show that the functionality of tropical forests can be maintained in moderately disturbed forest remnants. Conservation concepts for tropical forests should thus include the few remaining pristine forests [69] as well as functionally viable forest remnants that are also threatened by globally increasing land-use intensities and population numbers.

## Supporting Information

Figure S1 **Species turn-over among Biodiversity Observatories (BDOs) in six functional groups of animals in Kakamega Forest, Kenya.** Shown are ordination plots from non-metric multi-dimensional scaling (NMDS) on Bray-Curtis distances of the species abundances in each BDO; stress values of each NMDS are given. Effects of forest size on community composition were plotted onto the ordination plot if the relationship was significant (*P*<0.05). Filled symbols indicate BDOs situated in the main forest, open symbols in forest fragments. Malava East, Malava West and Kaimosi are much smaller in size than the other fragments; see Table S1 for exact forest sizes.
(EPS)

Table S1 **Forest size, intensity of selective logging, management authority and ecosystem processes studied in each of the 11 Biodiversity Observatories (BDOs) in Kakamega Forest, Kenya.** BDOs are ordered by forest size into study sites in forest fragments and main forest block; please refer to Fig. 1 for the exact location of each BDO. The intensity of selective logging was measured as the number of logged trees (with a DBH >10 cm) per ha during the last 20 years. Management authorities are Kenya Wildlife Service (KWS) or Kenya Forest Service (KFS); Kaimosi is privately owned and similarly managed as KFS sites. Ecosystem processes that were studied in the respective BDO are abbreviated as follows: po  =  pollination; sd  =  seed dispersal; sp  =  seed predation; de  =  decomposition; aa  =  army-ant predation; ab  =  antbird predation.
(DOC)

Table S2 **Relationships between human disturbance, species richness or community composition and ecosystem processes in Kakamega Forest.** The same path model was fitted for each ecosystem process affected by human disturbance (Fig. 3). Human disturbance increases from low to high values (forest size was multiplied by −1). Given are the number of study sites (in parentheses), standardized path coefficients, and their *P*-values from maximum-likelihood (ML) and parametric bootstrapping (boot) estimates. Bootstrap estimates are based on 1,000 iterations on non-standardized regression coefficients. Biodiversity effects were tested in terms of community composition (site scores of the first NMDS axis), except for bee species richness (see Table 2).
(DOC)
