# Supplementary material for: Forest Fragmentation and Selective Logging Have Inconsistent Effects on Multiple Animal-Mediated Ecosystem Processes in a Tropical Forest
Source: PLoS One. 2011 Nov 16;6(11):e27785. doi: 10.1371/journal.pone.0027785 (PMC3218041; doi:10.1371/journal.pone.0027785)
Supplement: Table S1 — Forest size, intensity of selective logging, management authority and ecosystem processes studied in each of the 11 Biodiversity Observatories (BDOs) in Kakamega Forest, Kenya. BDOs are ordered by forest size into study sites in forest fragments and main forest block; please refer to Fig. 1 for the exact location of each BDO. The intensity of selective logging was measured as the number of logged trees (with a DBH >10 cm) per ha during the last 20 years. Management authorities are Kenya Wildlife Service (KWS) or Kenya Forest Service (KFS); Kaimosi is privately owned and similarly managed as KFS sites. Ecosystem processes that were studied in the respective BDO are abbreviated as follows: po = pollination; sd = seed dispersal; sp = seed predation; de = decomposition; aa = army-ant predation; ab = antbird predation. (DOC) [file pone.0027785.s002.doc]

| BDO | Forest size [ha] | Number of logged trees [per ha] | Management authority | Ecosystem processes |
| --- | --- | --- | --- | --- |
| **Forest fragments** | |  |  |  |
| Malava East | 40 | 5.4 | KFS | po/sd/sp/de/aa/ab |
| Malava West | 78 | 10.0 | KFS | de/aa/ab |
| Kaimosi | 101 | 30.0 | private | po/sd/sp/de/aa/ab |
| Kisere | 406 | 8.5 | KWS | po/sd/sp/de/aa/ab |
| Yala | 1086 | 1.5 | KFS | po/sd/sp/de/aa/ab |
| Ikuywa | 1348 | 33.5 | KFS | po/sd/sp/de/aa/ab |
| **Main forest** |  |  |  |  |
| Buyangu | 9495 | 4.1 | KWS | po/sd/sp/de/aa/ab |
| Colobus | 9495 | 2.3 | KWS | po/sd/sp/de/aa/ab |
| Isecheno 1 | 9495 | 12.5 | KFS | po/sd/sp/de/aa/ab |
| Isecheno 2 | 9495 | 9.0 | KFS | po/sd/sp/de/aa/ab |
| Salazar | 9495 | 0.0 | KWS | po/de/aa/ab |
