# Supplementary material for: Forest Fragmentation and Selective Logging Have Inconsistent Effects on Multiple Animal-Mediated Ecosystem Processes in a Tropical Forest
Source: PLoS One. 2011 Nov 16;6(11):e27785. doi: 10.1371/journal.pone.0027785 (PMC3218041; doi:10.1371/journal.pone.0027785)
Supplement: Table S2 — Relationships between human disturbance, species richness or community composition and ecosystem processes in Kakamega Forest. The same path model was fitted for each ecosystem process affected by human disturbance (Fig. 3). Human disturbance increases from low to high values (forest size was multiplied by −1). Given are the number of study sites (in parentheses), standardized path coefficients, and their P-values from maximum-likelihood (ML) and parametric bootstrapping (boot) estimates. Bootstrap estimates are based on 1,000 iterations on non-standardized regression coefficients. Biodiversity effects were tested in terms of community composition (site scores of the first NMDS axis), except for bee species richness (see Table 2). (DOC) [file pone.0027785.s003.doc]

| Ecosystem process | Relationship | β | *P* (ML) | *P* (boot) |
| --- | --- | --- | --- | --- |
| Pollination | Logging – Pollination | 0.845 | <0.001 | 0.002 |
| (*N* = 10) | Forest size – Bee richness | 0.672 | 0.006 | 0.008 |
|  | Bee richness – Pollination | 0.085 | 0.629 | 0.678 |
| Seed dispersal | Logging – Seed dispersal | 0.813 | <0.001 | 0.010 |
| (*N* = 9) | Forest size – Frugivore community | 0.726 | 0.003 | 0.026 |
|  | Frugivore community – Seed dispersal | 0.285 | 0.112 | 0.197 |
| Decomposition | Logging – Decomposition | −0.288 | 0.219 | 0.290 |
| (*N* = 11) | Forest size – Leaf-litter community | 0.680 | 0.003 | 0.012 |
|  | Leaf-litter community – Decomposition | 0.608 | 0.009 | 0.021 |
| Army-ant raiding | Logging – Army-ant raiding | 0.636 | 0.009 | 0.048 |
| (*N* = 11) | Forest size – Army-ant community | 0.767 | <0.001 | 0.008 |
|  | Army-ant community – Army-ant raiding | −0.092 | 0.705 | 0.747 |
| Antbird predation | Logging – Antbird predation | −0.071 | 0.651 | 0.679 |
| (*N* = 10) | Forest size – Antbird community | 0.913 | <0.001 | 0.002 |
|  | Antbird community – Antbird predation | −0.880 | <0.001 | 0.002 |
